# He Korowai Manaaki (Pregnancy Wraparound Care): Protocol for a Cluster Randomized Clinical Trial

**DOI:** 10.2196/18154

**Published:** 2021-01-29

**Authors:** Beverley Lawton, Francesca Storey, Nokuthaba Sibanda, Matthew Bennett, Charles Lambert, Stacie Geller, Liza Edmonds, Fiona Cram

**Affiliations:** 1 Centre for Women's Health Research Victoria University of Wellington Wellington New Zealand; 2 Center for Research on Women & Gender, Center of Excellence in Women's Health Department of Obstetrics and Gynaecology University of Illinois Chicago, IL United States; 3 Women’s & Children’s Health University of Otago Dunedin New Zealand; 4 Katoa Ltd Auckland New Zealand

**Keywords:** maternity, inequity, Indigenous health, Māori, pregnancy, Kaupapa Māori, socioeconomic, primary health care, methodology

## Abstract

**Background:**

Maternal and infant health inequities between Māori (the Indigenous peoples of Aotearoa New Zealand) and New Zealand European women are well documented and cannot be explained solely by socioeconomic status. A research center-iwi (tribal group) partnership aims to address these disparities and improve maternal and infant health outcomes by implementing an augmented maternity care pathway (He Korowai Manaaki) to improve access to services and evidence-informed care.

**Objective:**

The objective of this study is to test whether an augmented maternity care pathway improves Māori infant health outcomes.

**Methods:**

This is a Kaupapa Māori (by, with, and for Māori) cluster randomized clinical trial involving 8 primary care practices allocated to either an intervention arm or control arm. The intervention arm comprises an augmented maternity care pathway (He Korowai Manaaki) offering clinical care through additional paid health care appointments and improved access to social support (eg, housing, transport). The control arm is usual care. The primary outcome is increased timely vaccination for Māori infants, defined as all age-appropriate vaccinations completed by 6 months of age.

**Results:**

Recruitment commenced in November 2018 and was completed in June 2020, with 251 enrolled women recruited in intervention primary care practices before 20 weeks of pregnancy. Publication of results is anticipated in late 2023.

**Conclusions:**

The results will inform primary health care policy including whether the provision of augmented maternal care pathways reduces disparities in the structural determinants of health. If effective, He Korowai Manaaki will strengthen the health and well-being of pregnant Māori women and their babies and improve their health outcomes, laying a strong foundation for lifelong health and well-being.

**Trial Registration:**

Australian New Zealand Clinical Trials Registry ACTRN12619001155189; https://tinyurl.com/yypbef8q

**International Registered Report Identifier (IRRID):**

DERR1-10.2196/18154

## Introduction

### Background

Protecting the health and well-being of expectant mothers and their families helps ensure that they and their baby/ies are well cared for and supported to have good maternal outcomes. In Aotearoa New Zealand, Indigenous Māori women have higher rates of adverse pregnancy outcomes compared to non-Māori women. Māori infants have an infant death rate of 5.9 per 1000 births compared to 3.2 per 1000 births among non-Māori [1]. Māori pregnant women and children also experience substantial socioeconomic disadvantages. Even so, the health inequities between Māori and New Zealand European women and infants are well documented and cannot be explained solely by socioeconomic status [2]. Reducing these health and socioeconomic disparities is an urgent priority.

At the invitation of the iwi (tribal group), He Korowai Manaaki (a protective cloak) was designed to surround pregnant woman and their children with the best evidence-informed, timely care and the best environment. As an augmented maternal care pathway, He Korowai Manaaki was designed to improve health and well-being through pregnancy and baby’s first 2 years of life and beyond.

Aotearoa New Zealand’s unique midwifery-led model of maternity care, established nearly 30 years ago, was purported to hold the potential to improve health outcomes for Māori [3]; however, Māori women and whānau (family) continue to experience persistent health inequities that impact well-being throughout the maternity continuum [4]. Underrepresentation of Māori midwives at all levels of the profession is indicative of a colonized infrastructure with Māori childbirth knowledge treated with skepticism [4].

Most women begin their pregnancy journey with their primary health care provider; however, the current model does not easily support continued primary health care practice involvement during pregnancy nor postpartum. Transitioning to a lead maternity carer (LMC; typically a midwife) can lead to fragmented, siloed care, inhibiting a seamless pregnancy pathway [2] and potentially contributing to health disparities for Māori women and babies [5].

Therefore, changes in the structure of the maternity health system are required, as improved coordination between midwives and general practitioners could be of great benefit for both equity of outcomes and efficiency of health service delivery [6].

### Iwi Partnership

The research center Te Tātai Hauora O Hine (The Centre for Women’s Health Research, Victoria University of Wellington) was invited to partner with iwi/tribal group Ngāti Pāhauwera and develop a wraparound approach to maternity care. The aim of this approach was the provision of a seamless maternal and infant care pathway with improved access to both clinical and social support [7].

The guiding principles of the Ngāti Pāhauwera strategic plan are *Pakatō i te ata, Pakatō i te ahiahi, Māuri mahi Māuri ora* (planning for the future health and well-being of the people), and *Mahia nga māhi o Kahukura* (imagining and creating a better future). These guiding principles provide foundations for the research partnership [7] and the resulting He Korowai Manaaki pathway (ACTRN12619001155189).

Grounded in a Kaupapa Māori (by, with, and for Māori) inquiry paradigm, this research prioritizes Māori ways of knowing and being, promotes a structural analysis of inequality [8], and aims to benefit Māori through the reduction of disparities [8-10]. The research practices reflect tikanga Māori (Māori customs), including the importance of place, relationships, and Māori self-determination [8,9].

### Aims

This study aims to implement an augmented maternity care pathway (He Korowai Manaaki) to improve Māori maternal and child health outcomes and to improve access to services (health, education, Well Child Tamariki Ora [WCTO], oral health, contraception, general practice) for pregnant Māori women and their infants.

We hypothesize that the He Korowai Manaaki pathway, with early, evidence-informed care and ongoing wraparound support opportunities, will improve the health outcomes of Māori infants. If successful, this pathway will serve as a prototype for an augmented national maternity care pathway.

## Methods

### Design

This study is a cluster randomized clinical trial with 2 study arms for pregnant women enrolled with primary care practices (PCPs). Practices are the unit of randomization. Intervention PCPs utilize the He Korowai Manaaki pathway for the pregnant women in their practice. Control PCPs continue usual care. For collection of data, all women in the intervention are individually consented, in contrast to the control arm where deidentified data is collected without individual consent.

### Research Approval

Research ethics approval was granted by the Health and Disability Ethics Committee of New Zealand (17/STH/136) in August 2017.

### Study Sites

All 15 PCPs in the urban Hawkes Bay region of Aotearoa New Zealand were approached; 8 provided informed written consent to participate in the cluster randomized clinical trial and were randomized: 4 to intervention and 4 to control. All pregnant women enrolled as a patient of an intervention PCP are eligible for the intervention.

### Sample Size

A total of 8 practices and 216 Maori participants (4 practices and 108 Maori participants in each group) will provide 80% power at a two-sided α of .05 to detect an 18.5% difference in the proportion of infants who receive all age-appropriate vaccinations by 6 months of age between the groups. For these figures, we have assumed an average cluster size of 27, intracluster correlation coefficient of 0.01, and that 83.5% of the infants will receive all age-appropriate vaccinations by 6 months of age in the intervention group and 65% in the control group. Our total sample size requires 432 pregnancies (216 control, 216 intervention).

Based on the expected recruitment rate and pregnant women meeting the entry criteria for the core intervention (ie, seen in a PCP before 20 weeks of pregnancy), He Korowai Manaaki is offered through intervention practices to all pregnant women for approximately 18 months from the commencement of the study to obtain the required sample size.

### Randomization

Practices are the unit of randomization. Each of the 8 PCPs was randomly allocated to either the intervention arm (He Korowai Manaaki) or the control arm (usual care) of the trial. Covariate constrained randomization [11,12] was used to minimize potential imbalance between intervention and control arms in the size of the Māori population aged less than 1 year. Information on enrollment size and numbers of Māori patients aged less than 1 year was collected for each of the 8 participating PCPs. All possible allocations of these PCPs to the 2 trial arms were then enumerated using an algorithm blinded to the practice names. The list of allocations was then narrowed down to the ones that gave approximate balance in the numbers of Māori aged less than 1 year across the 2 arms, with each arm having 4 clusters (PCPs). Finally, the actual allocation was chosen randomly from this constrained list, thereby achieving an acceptable allocation while retaining randomness in the selection process. No practices dropped out of the study.

### Control Arm (Usual Care)

Women enrolled in control practices who are pregnant during the study period receive usual care and will be included as controls in the trial. In Aotearoa New Zealand, usual care means that a woman chooses their LMC, and for most women, their LMC is an independent or self-employed midwife [13]. A high proportion of the control cohort is likely to have engaged with primary care early in pregnancy but continued primary health care involvement during pregnancy and postpartum is unlikely. Usual care for the control cohort is expected to be predominantly midwifery-led pregnancy care.

### Intervention Arm (He Korowai Manaaki)

The He Korowai Manaaki intervention addresses both clinical care (pregnancy, postpartum, neonatal, and reproductive health) and the structural determinants of health (eg, housing, transport) with a best-practice pathway. He Korowai Manaaki includes responses to recommendations from the New Zealand Perinatal and Maternal Mortality Committee [14] and is in line with recommendations to address structural determinants of health by the Select Health Committee Inquiry into Improving Child Health Outcomes [15]. As a practice service change, He Korowai Manaaki is facilitated through primary care–held appointments including an extended first visit (First Touch), a follow-up visit, a third trimester visit, and a 6-week postnatal whānau (family) visit. Usual lead maternity care continues throughout.

General practitioners, nurse practitioners, and practice nurses at intervention PCPs were asked to attend an introductory training session on He Korowai Manaaki to enable the practice service change. The in-practice session provided by the researchers included education refreshers of evidence-based antenatal care, postnatal care, and contraception as well as information on the 4 study-funded appointments and utilization of the pregnancy wraparound care computerized advanced form installed into their practice management system. Education refresher sessions are provided by the researchers and associated experts over the course of the trial.

Clinicians in intervention PCPs work from the computerized advanced form to support care, screening, and navigation to allied services. This includes referrals to specialist care and services meeting the needs of wraparound care (eg, housing program, social work services, driving licensing programs, and dental practices).

Intervention practices are also supported to provide contraception of the woman’s choice, free of charge (see third trimester appointment and 6-week postnatal appointment in Textbox 1) and support with transport to pregnancy-related appointments (see First Touch appointment in Textbox 1).

Posters and published material on display at each intervention practice inform the enrolled population of the He Korowai Manaaki practice change taking place for the duration of the research project. All women identifying as pregnant in a primary care appointment are informed that their practice is offering an augmented pathway of care for pregnant women as part of the research project.

Information about the project is also shared with individual women by the general practitioner, nurse practitioner, or practice nurse. Each woman is asked to provide informed written consent for their deidentified outcome data (pregnancy and infant health information) to be shared with the research group in the future. The augmented pathway visits (Textbox 1) are explained, with the First Touch appointment then being offered to all women (with no data being shared for nonconsenting women). All women are then invited to attend the other study appointments (free of charge), and a recall for the next appointment is set.

There are no exclusions. The augmented pathway of care is available at any stage of pregnancy, for all pregnancies (low-risk and high-risk).

**He Korowai Manaaki appointment descriptions.**

First Touch appointment **-** an extended first antenatal appointment that includes:

- Time taken to respond fully to concern and queries
- Offer of screening for congenital abnormalities, sexually transmitted infections, family violence, and maternal mental health, with referrals as warranted
- Diagnosis of any underlying medical conditions, with referral to secondary care as appropriate
- Identification of risks (maternal age, obesity, maternal mental health problems, multiple pregnancy, socioeconomic deprivation, maternal medical conditions, previous preterm deliveries) with referral to secondary care as appropriate
- Navigation to lead maternity carer (LMC)
- Offer of prescribed pregnancy medications (folic acid, iodine)
- Whānau (family) checklist to assess whether support is required for transport to appointments, safe housing, finance, and oral health with connection offered to existing services and support

Follow-up appointment, including:

- Follow-up of tests that have been ordered, making sure all appropriate referrals have been made
- Ensure enrollment with LMC
- Administration of maternal vaccinations, when appropriate

Third trimester appointment (open to woman’s midwife or whānau [family] to attend), including:

- Maternal vaccinations and planning for infant(s), including the provision of best-practice information about maternity health, child health
- Conversation about and planning for postnatal contraception

6-week postnatal appointment (open to woman’s midwife or whānau [family] to attend), including:

- Addressing any concerns and answer queries
- Provision of free contraceptive
- Screening for infections, family violence, and maternal mental health, with referrals as appropriate
- Education around nutrition, smoking, alcohol use, and drug use
- Education around pelvic health, navigation to women’s physio service as appropriate
- Navigation to oral health care services
- Navigation to support services such as Family Start, Well Child/Tamariki Ora, and Early Childhood Education services

### Primary Outcome Measures

The primary outcome is the increase in timely vaccinations for Māori infants. Timely vaccination is defined as all age-appropriate vaccinations completed by 6 months of age.

### Secondary Outcome Measures

Secondary outcomes include infant hospitalizations and length of stay until 1 year of age as well as obstetric, delivery, and infant outcomes plus service engagement outcomes (contraception, oral health, WCTO, Early Childhood Education [ECE]; see Table 1).

**Table 1:** Outcome variables and data sources.

Data source	Data source description	Examples of outcome variables
ELI^a^	The Ministry of Education information system for Early Childhood Education (ECE) collects information on participating children's enrollment and attendance.	Infant registration with ECE/Te Kōhanga Reo at 2 years of age^b^
MAT^c^	The MAT provides statistical, demographic, and clinical information about selected publicly funded maternity services up to 9 months before and 3 months after a birth. It also contains inpatient and day-patient health event data on pregnancy, birth, and the postnatal period for mother and baby, sourced from the National Minimum Dataset (administered by the MOH^d^).	Maternal ethnicity; smoking status at time of booking with a maternity care provider and at 2 weeks postdelivery^b^; hospitalization episodes^b^; antenatal screening^b^; plurality; parity; mode of delivery^b^; Apgar scores^b^; birthweight; gestational age at delivery^b^; infant hospitalization in first year of life^b^; breastfeeding status at infant discharge^b^, 2 weeks^b^, and 6 weeks
MOH	The MOH receives data from different parts of the health sector through the utilization of health services or mandatory reporting national collections and also from national population health surveys.	Infant registration to oral health services at 2 years of age^b^
MORT^e^	The MORT classifies the underlying cause of death for all deaths registered in New Zealand and all registerable stillbirths using the World Health Organization Rules and Guidelines for Mortality Coding.	Infant mortality^b^, ethnicity, date of death, gestational age at death, birthweight, diagnostic codes on cause of death, sudden and unexpected death indicator
NIR^f^	The NIR is a computerized information system that has been developed to hold immunization details of New Zealand children (administered by the MOH).	Infant vaccination at 6 weeks^g^, 3 months^g^, 5 months^g^, and 15 months^b^
NHI^h^	The NHI is a system used by public hospitals and other health and disability support services to assign an alphanumeric identifier (the NHI number) to people who use their services.	NHI number, area deprivation, ethnicity (maternal and infant)
NMDS^i^	The NMDS is a national collection of public and private hospital discharge information, including coded clinical data for inpatients and day patients (hospital events).	Ethnicity; diagnostic codes (ICD-10^j^)^b^; maternal, antenatal, or postnatal hospital admissions (public)^b^; discharge dates and length of stay; infant hospital admissions (public)^b^; discharge dates and length of stay
PHO^k^	The PHO provides a national enrollment collection that holds patient enrollment data.	Infant engagement with general practitioner <8 weeks postdelivery or after infant discharge from hospital^b^
WCTO^l^	The WCTO program is a series of health assessments and support services for children and their families from birth to 5 years and is a gateway for parents to access primary and specialist health care, education, and social services. WCTO providers submit service coverage and data to the MOH.	Attendance at scheduled WCTO infant appointments at 8-10 weeks^b^, 3-4 months^b^, 5-7 months^b^, 9-12 months^b^, and 15-18 months^b^; breastfeeding status at 3 months and 6 months^b^

^a^ELI: Early Learning Information System – Ministry of Education.

^b^Secondary outcome.

^c^MAT: National Maternity Collection.

^d^MOH: Ministry of Health.

^e^MORT: Mortality Collection.

^f^NIR: National Immunisation Register.

^g^Primary outcome.

^h^NHI: National Health Index.

^i^NMDS: National Minimum Dataset.

^j^ICD-10: International Classification of Diseases, Tenth Revision.

^k^PHO: Primary Health Organisation.

^l^WCTO: Well Child/Tamariki Ora.

### Data Collection

The data for our study are generated from national and local information collections, as described by Filoche et al [16]. In Aotearoa New Zealand, the Ministry of Health (MOH) is responsible for the oversight and funding of the country’s 20 district health boards. Select clinical information is routinely reported by each health board to the MOH and is collated into national datasets with operational responsibility by the Client Insights and Analytics group. Outcome data will be collected using Aotearoa New Zealand’s unique patient National Health Index (NHI) number to source clinical and demographic data from multiple national datasets (Table 1).

**Figure 1:**
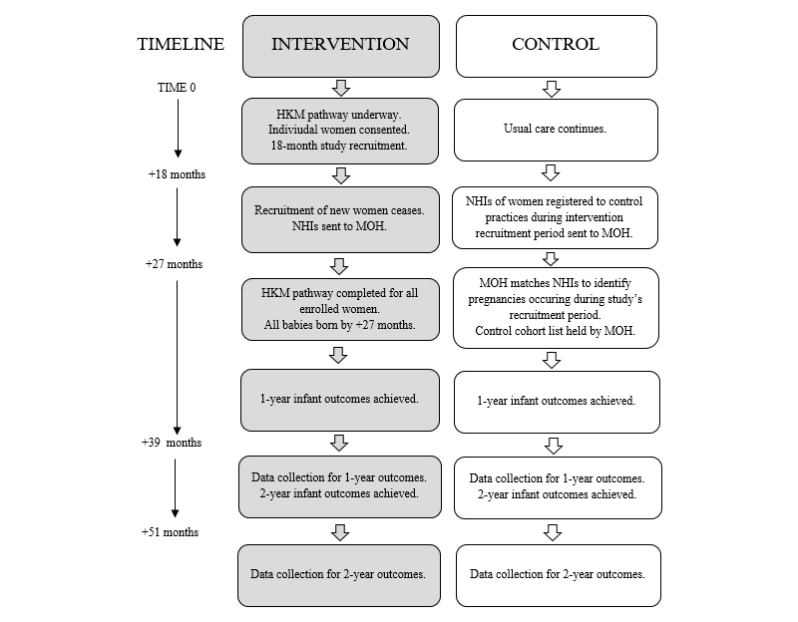
He Korowai Manaaki (HKM) timeline. MOH: Ministry of Health; NHI: National Health Index.

At study end, intervention practices will send the NHIs of women who have provided consent for their deidentified outcome data to be collected and analyzed (linked to the NHI of an infant) to the MOH. The MOH will also receive from each control practice the NHIs of women registered with them during the study’s recruitment period. To identify the control group (women enrolled with PCPs, pregnant during the study period) and the associated linked infant(s) following the relevant designated time period (Figure 1), the MOH will match the NHIs provided by the control practices to national datasets containing maternity, pregnancy, and delivery information (eg, the National Maternity Collection and the National Minimum Dataset).

### Study Variables

Primary and secondary outcomes will be tracked by patient NHI without names. The NHIs will be matched to multiple national databases (Table 1) up until the infant is 2 years of age to source clinical and demographic data to provide a combined data source for (1) sociodemographic information (ethnicity, New Zealand Index of Deprivation [socioeconomic status], maternal age), (2) clinical information (parity, plurality, LMC, gestational age at booking), (3) obstetric outcomes (gestation at delivery, cesarean section, Apgars, mortality, and morbidity), (4) antenatal screening (LMC registrations), (5) smoking status (maternal smoking status at booking and after delivery), (6) vaccination status (timely access to age-appropriate immunizations), (7) infant hospitalizations (cause of mortality, intraventricular hemorrhage [bleed in brain], oxygen required on discharge, and length of stay in neonatal intensive care unit), (8) access to child health services (number of oral health or WCTO visits, general practice registrations), and (9) access to ECE (ECE registrations).

### Analysis

Data analysis will occur as soon as outcome data are available for the intervention and control arms. Within a Kaupapa Māori inquiry paradigm [8-10], the primary analysis is for Māori, with secondary analysis for non-Māori [17]. An intention-to-treat analysis will be undertaken [18] using individual participant data. All pregnancies will be analyzed (regardless of the number of He Korowai Manaaki appointments attended), with the primary cohort being women seen in a PCP before 20 weeks of pregnancy.

The rate of infant hospitalizations will be analyzed using Poisson regression, and if there is evidence of overdispersion or underdispersion, then negative binomial model will be used. A generalized linear mixed model with a logit link will be used to analyze binary outcomes, and a linear mixed model will be used to analyze continuous outcomes. All regression models will adjust for potential personal and care-related confounders and for the effect of clustering within practices. Sensitivity analyses will be undertaken for the primary outcome to determine the impact of missing data, and per protocol analyses will be conducted. The consistency of effects for prespecified subgroups will be assessed using tests for heterogeneity.

Descriptions of rates, rate ratios, odds ratios, and respective 95% confidence intervals will be reported. Results will be aggregated, and no individual practice will be identifiable.

## Results

This cluster randomized clinical trial is underway with 8 PCPs. Practices have been randomized to either the intervention arm or control arm. Recruitment of women ended in June 2020, with 293 women enrolled in the intervention arm, of which 251 women (the primary cohort) were seen in a PCP before 20 weeks of pregnancy. Data collection will commence in early 2022 and be complete by mid-2023, and the analysis results are anticipated to be published in late 2023. The explicit and conscious decision to use an indigenous lens when analyzing the data allows outcomes to be viewed with a focus on advantage and privilege rather than one of disparity.

## Discussion

Quality, culturally responsive maternal care is expected and essential to the achievement of Māori pregnancy, birthing, and motherhood aspirations of “hapū ora” [19], that is, the health and well-being of Māori mothers-to-be and their babies. Pregnancy is an important period during which health and support services can provide information, care, and resources to enable the optimal environment for fetal and neonatal stages of life [19]. Quality antenatal care is especially important as part of a continuum of health care for mothers and as the starting point for the child’s developmental trajectory [20]. Māori women have a higher prevalence of maternal risk factors compared to other women and have greater maternity needs [21]; yet, their access to maternal health care and social support does not reflect this. As primary maternity care is considered to be a key enabler of health and well-being, it is pivotal that we find structural solutions that support hapū ora [19].

This augmented pathway enabled through primary care aims to achieve equitable outcomes by meeting the structural determinants and health needs of pregnant Māori women. If successful, the findings of this trial will inform policy makers and service providers to bring about system changes.

### Limitations

The study limitations include whether PCPs find He Korowai Manaaki useful in their practice. Given the heterogeneous make-up of PCPs, the benefits for each practice may vary depending on their population, resources, and needs. The study does not measure which component(s) of the pathway are taken up; however, an intention-to-treat analysis is widely accepted as the gold standard for assessing the superiority of the intervention in randomized trials [18].

Further, this study will be carried out in an urban area with a high Māori population, and the results may not be generalizable to other areas and other communities.

### Conclusions

The results of this study will inform policy and clinical pathways for Māori and be valuable in informing agencies about the potential health and well-being gains from an iwi-initiated augmented national maternity care pathway accessible through primary care.

## Abbreviations

ECE: Early Childhood Education
LMC: lead maternity carer
MOH: Ministry of Health
NHI: National Health Index
PCP: primary care practice
WCTO: Well Child Tamariki Ora
